# Tezepelumab for severe asthma: elevating current practice to recognize epithelial driven profiles

**DOI:** 10.1186/s12931-024-02998-6

**Published:** 2024-10-09

**Authors:** Marco Caminati, A. Vatrella, P. Rogliani, E. Carpagnano, A. Spanevello, G. Senna

**Affiliations:** 1 Allergy Unit and Asthma Center, Integrated University Hospital of Verona, Verona, Italy; 2 Department of Medicine, University of Verona, Verona, Italy; 3 Department of Medicine, Surgery and Dentistry, University of Salerno, Salerno, Italy; 4 Unit of Respiratory Medicine, Department of Experimental Medicine, University of Rome “Tor Vergata”, Rome, Italy; 5 Respiratory Diseases Section, Department of Basic Medical Science Neuroscience and Sense Organs, University of Bari, Bari, Italy; 6 Istituti Clinici Scientifici Maugeri IRCCS, Tradate , University of Insubria, Varese, Italy

**Keywords:** Biomarkers, Epithelial barrier, Phenotyping, Severe asthma, Tezepelumab

## Abstract

**Background:**

An increasing amount of evidence supports the relevance of epithelium across the wide spectrum of asthma pathobiology. On a clinical ground tezepelumab, selectively binding TSLP, a major epithelial cytokine, has demonstrated to be effective in asthma patients regardless their specific phenotype. In order to avoid the risk of considering tezepelumab as a not-specific option, the present perspective aims to sketch the tezepelumab best eligible patient profile and to propose some hallmarks of epithelial-driven disease by reviewing the published evidence on the drug mechanism of action and efficacy data.

**Main body:**

Although it cannot rely on standardised or exclusive “markers”, the relationship between environment and poor asthma control might suggest a major relevance of the epithelial barrier dysfunction. In that light, allergy and asthma exacerbations concomitant with specific exposures (pathogens, pollutants, chemicals), as well as increased susceptibility to infections can be considered as the hallmark of an impaired epithelial immune response. Tezepelumab is effective in allergic patients, being able to reduce asthma exacerbations precipitated by the exposure to seasonal or perennial aeroallergens, including fungi. In addition, tezepelumab reduced the incidence of co-occurring respiratory illness and asthma exacerbations. In terms of inflammation, epithelial immune response has been related to an impaired mucus hypersecretion and plugging. A placebo-controlled trial demonstrated a significant reduction of mucus plugging in treated patient. Airways hyperreactivity (AHR), airways obstruction and remodelling have been described as an expression of epithelial orchestrated immunological activation. Of note, a significantly higher incidence of mannitol negative test in patients treated with tezepelumab when compared to placebo group has been observed. In addition, A 130 mL improvement in pre-BD FEV1 has been described in patients assuming Tezepelumab. The above-mentioned data suggest that bronchial reversibility and AHR can be considered “functional biomarkers” supporting patients’ phenotyping and the identification of tezepelumab best responders.

**Conclusion:**

Integrating “functional biomarkers” to the inflammatory ones and a better characterization of asthma exacerbations might pave the way to a different and more transversal phenotyping, which overcomes the “restrictive” labels including T2 high, allergic/atopic or T2 low asthma. Precisely defining the disease characteristics and potential targets for a better control even in tezepelumab eligible subjects is essential to avoid the block buster temptation and optimize the personalized medicine approach according to each patient’s individuality.

## Background

The central role of epithelium across the wide spectrum of asthma phenotypes and endotypes has been clearly demonstrated in the last few years [1]. In asthma patients, epithelial cytokines orchestrate the initiation and persistence of an unbalanced response leading to airways chronic inflammation. Thymic stromal lymphopoietin (TSLP) is particularly involved at the top of and throughout the immune cascade in response to environmental and pro-inflammatory stimuli [1]. Whether the underlying inflammation is predominantly allergic, eosinophilic or non T2, such impaired interaction between host and environment represents a common pathobiological background [2]. Within that frame, by selectively targeting TSLP tezepelumab has demonstrated to be extremely effective in every asthma patient [3]. When reviewing the recently proposed algorithms supporting the treatment selection process in severe asthma patients eligible to biologics, all of them include tezepelumab as a valuable option for every type of asthma [4, 5]. It’s not surprising nor incorrect in the light of tezepelumab mechanism of action and according to the evidence of its efficacy so far [3]. On the other side looking at tezepelumab as a “block-buster” option wouldn’t be consistent with the modern precision medicine approach aiming to tailor the overall asthma management according to each patient’s individual profile. In addition, a number of different monoclonal antibodies targeting T2 cytokines are currently part of severe asthma therapy armamentarium, a robust amount of evidence supporting their full efficacy and safety [4]. This scenario cannot be neglected and further sustains the need for precisely defining the place for tezepelumab.

The aim of the present perspective is to review the published evidence on tezepelumab mechanism of action and efficacy data in order to sketch the tezepelumab best eligible patient profile and to propose some hallmarks of epithelial-driven disease. A literature search of the PubMed database was undertaken to identify papers published in indexed journals up to May 2024 according to the following research keywords: tezepelumab, severe asthma AND tezepelumab, chronic rhinosinusitis with nasal polyps (CRSwNP) AND tezepelumab. The search results were integrated with additional literature identified ad hoc or via the bibliographies of identified studies. Case reports, correspondence, editorials, and non- English language articles were excluded. Papers that were considered more informative and aligned with the purpose of the review according to the authors judgement were retained.

## Main text

In Europe and US severe asthma patients are eligible to tezepelumab if they experienced “asthma exacerbations in the previous year”. No additional criteria are mentioned by the regulatory requirements [6, 7]. In the light of the heterogeneous spectrum of asthma pathobiology, such a vague indication doesn’t support the best responder patient identification, which would benefit from a combined evaluation of the patient’s profile and the evidence of specific drug-related outcomes.

When reviewing the scientific literature besides the regulatory requirements, tezepelumab is recommended in the case of not eligibility or poor response to other currently marketed treatments for severe asthma [4, 5]. That approach is undoubtfully consistent, but prescribing anti-TSLP by exclusion or as a second line treatment only might be quite restrictive, and unreasonably delay the prescription of the more appropriate treatment option. According to the current clinical practice, asthma phenotyping mainly relies on inflammation biomarkers, including blood eosinophils, total and specific IgEs, and exhaled nitric oxide (FeNO). In fact, eosinophilic, allergic, or more generically T2 high asthma is defined in relation to their differential expression. If none of them can be detected, the disease is classified as non T2, or T2 low [8]. In the last case, tezepelumab is at the moment the only option, due to the lack of eligibility to all the other currently available biologics [6, 7]. On the opposite, T2 high asthma represents a much more complex scenario. In fact, each one of the marketed monoclonal antibodies, including tezepelumab, showed to be effective in the presence of raised T2 biomarkers [4, 5].

Of note, the coexistence of more than one T2 biomarker, namely BEC ≥ 300 cells per microliter and IgE sensitization with concomitant elevated FeNO values, has been associated with a higher response to tezepelumab. It suggests that patients demonstrating an allergic or eosinophilic phenotype and characterized by multiple inflammation drivers, could relevantly benefit from anti-TSLP. [9, 10]. In particular, a significant exacerbation rate reduction, lung function improvement and better overall asthma control has been reported in that population when treated with tezepelumab [3, 9].

According to a more personalized approach, biological markers can be contextualized within the clinical characteristics of the disease, including at least exacerbation triggers, disease onset, response to standard treatment, lung function and airway hyperreactivity, comorbidities. Furthermore, the identification of an epithelial driven disease might also contribute to profile the patient’s phenotype, in combination with the traditional inflammatory biomarkers, especially when they not univocally orient to one specific treatment.

Although at the moment it cannot rely on any standardised or univocal “marker”, a major relevance of the epithelial barrier dysfunction might be suggested by the relationship between environmental determinants and poor asthma control. In that light, increased susceptibility to infections and asthma exacerbations concomitant with specific exposures (pathogens, pollutants, chemicals.) could represent an evidence. Similarly, allergy and perhaps sensitization, inducing both IgE and eosinophilia, can be considered as the hallmark of an impaired, epithelial mediated, interaction between the environment and the immune system [1]. Tezepelumab efficacy in sensitized asthma patients has been clearly demonstrated [9], although in the presence of some potential bias. In fact, most of data regarding tezepelumab and allergic asthma have been generated looking at sensitization only, but the specific relevance of total and specific IgE when not matched to clearly related clinical manifestations is actually unclear. However, in tezepelumab treated patients, a significant reduction of asthma exacerbations precipitated by the exposure to specific seasonal or perennial aeroallergens have been observed [11]. In the case of perennial allergen sensitization, overlapping eligibility with omalizumab has to be evaluated. However, tezepelumab might represent the first choice when asthma exacerbations predominantly occur in concomitance with the allergen peaks, or when other environmental triggers (i.e. pathogens) besides allergens lead to poor asthma control. In addition, when considering some peculiar allergens, including fungi, Alternaria and house dust mites, the IgE mediated cascade is not the only one mechanism activated by the environmental exposure. In fact, they can exert a direct tissue damage through their proteases, and interact with innate immunity receptors (pathogen recognition receptors, toll-like receptors), overall amplifying the epithelial susceptibility and impairment. Those mechanisms lead to an “allergic” and eosinophilic inflammatory response even in the absence of specific IgE sensitization [12]. Of note, a recent post-hoc analysis has demonstrated tezepelumab efficacy in patients with severe uncontrolled asthma and fungal sensitization with regard to exacerbation rate, lung function and patient reported outcomes [13]. Further real-life evidence, maybe including patients clearly experiencing asthma worsening associated with exposure to fungal allergens (e.g. allergic bronchopulmonary aspergillosis patients), is needed to support tezepelumab relevance in the management of that specific severe asthma phenotype. However anti-TSLP mechanism of action combined with the post hoc analysis data mentioned above provide a strong rationale for the use of tezepelumab in severe asthma with fungal sensitization.

Viral and bacterial acute respiratory infections can trigger increased bronchial epithelial expression of TSLP and cause asthma exacerbations. And, on the other way round, a disrupted epithelium predisposes to a higher susceptibility to infections [14]. More in details, a long form of TSLP is typically produced by epithelial cells of asthma patients following viral infection. It causes a decreased IgA antibodies production and impacts on the neutralisation of viruses at mucosal surfaces. Respiratory infections result in a disruption of junctional complexes and impaired repair processes, making the epithelial barrier more susceptible. Not surprisingly, according to a post-hoc analysis of PATHWAY and NAVIGATOR Tezepelumab was able to reduce the incidence of co-occurring respiratory illness and asthma exacerbations versus placebo. Although a specifically designed study would consolidate those findings, the results support the benefits of tezepelumab in severe asthma patients experiencing respiratory illnesses (i.e. cold, flu etc.) as a prominent asthma exacerbation trigger [15].

Mucus hypersecretion and plugging has also been related to an impaired epithelial immune response, as a consequence of goblet cell hyperplasia and mucin over-expression. Cough with phlegm, wheezing, dyspnoea, both in the context of acute exacerbations and as chronic manifestations, represent the clinical counterpart of mucus plugging in the airways, that can be quite easily detected by a high-resolution pulmonary CT scan. Not surprisingly, a placebo-controlled trial demonstrated a significant reduction of mucus plugging in patient treated with tezepelumab, in correlation with improvements in BECs, FeNO levels and FEV1, and reductions in IL-5 and IL-13 level [16].

As a further consequence of the epithelial immunological activation, the recruitment of mast cells, basophils and myofibroblast has been described [1, 14]. The first do contribute to bronchial hyperreactivity; mast-cells and basophils interact with bronchial smooth muscle, and myofibroblasts are major player of the remodelling processes. Under that perspective, bronchial hyperreactivity and FEV1, as a measure of bronchial obstruction and a proxy of bronchial remodelling, might be considered as functional hallmarks of an epithelial driven impairment. In addition, UPSTREAM and CASCADE phase 2 trials have demonstrated a significantly higher incidence of mannitol negative test among patients in the active arm when compared to the placebo group [17, 18]. A 130 mL improvement in pre-BD FEV, representing a bigger variation when compared to the minimal clinically important difference [19], has been observed in patients assuming tezepelumab vs. placebo [20]. According to a recent post-hoc analysis of phase 3 NAVIGATOR study, the functional change in treated patients is even more evident when a FEV1 reversibility > 20% is detected at baseline, [21]. Overall, those findings suggest that bronchial reversibility and AHR can be considered as kind of functional biomarkers supporting patients’ phenotyping and that a detailed lung function assessment might help in identifying tezepelumab best responders. On a practical ground, AHR might be hard to demonstrate, especially in asthma patients characterized by severely compromised lung function assessment; however, some suggestions can be derived by the patient’s history (i.e. cough or bronchospasm when exposed to environmental irritants). Furthermore, in the case of a negative bronchodilator response, a reversible bronchial obstruction might be demonstrated by repeating spirometry assessment immediately after an oral corticosteroid trial.

Asthma severity is strictly connected with the burden of its comorbidities, CRSwNP representing the most frequent and impactful one [22]. Recent translational evidence has reframed nasal polyps’ pathobiology within the epithelial barrier dysfunction perspective, which also accounts for its high frequency among severe asthma patients [23]. A focused analysis of tezepelumab efficacy on CRSwNP is still under investigation but not surprisingly it has been demonstrated that in treated patients’ asthma outcomes are not influenced by concomitant CRSwNPs, and a significant Sinonasal Outcome Test score reduction has also been observed [24].

## Conclusions

**Fig. 1:**
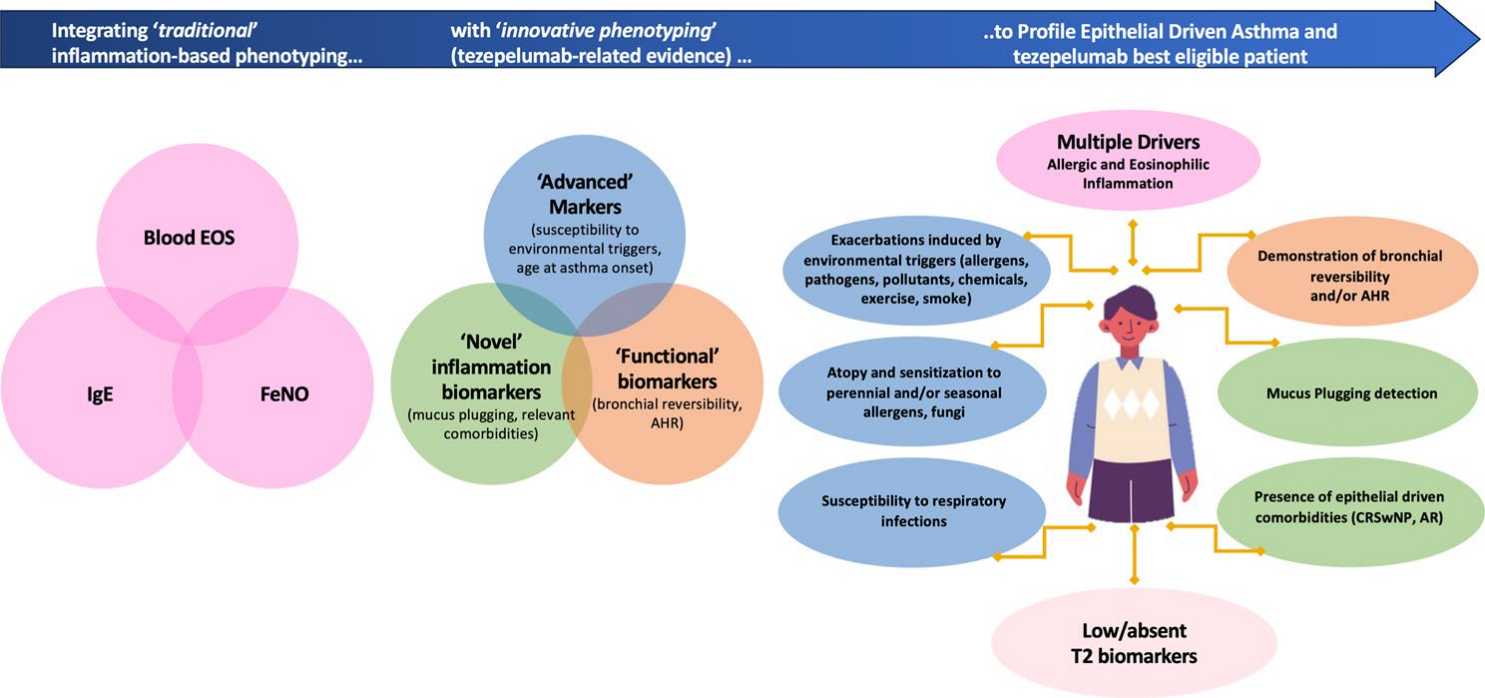
Proposed hallmarks of epithelial driven disease and markers of best eligibility to tezepelumab. AR: allergic rhinitis; AHR: airways hyperreactivity; CRSwNP: chronic rhinosinusitis with nasal polyps

Taken together, the available evidence on tezepelumab sustains its efficacy on each one of the three main asthma pathobiological features, including inflammation, whether allergic, eosinophilic or T2 low, bronchial obstruction and AHR. In addition, by directly targeting the epithelial barrier dysfunction, anti-TSLP showed an optimal efficacy profile in terms of “environmental driven” exacerbations. The evidence on specific drug-related outcomes contributes to define the best responder profile when matched with the patient’s peculiar characteristics (Fig. 1).

Furthermore, integrating “functional biomarkers” to the inflammatory traditional ones might pave the way to a different and more transversal phenotyping, which overcomes the “restrictive” labels including T2 high, allergic/atopic or T2 low asthma (Fig. 1). Under that perspective, focusing more on exacerbations aetiology, if related to environmental triggers or to endogenous proinflammatory stimuli, may further contribute to asthma phenotyping. Precisely defining the disease characteristics and potential targets for a better control even in tezepelumab eligible subjects is essential to avoid the block buster temptation and optimize the personalized medicine approach according to each patient’s individuality.

## Data Availability

No datasets were generated or analysed during the current study.

## Abbreviations

AHR: Airways hyperresponsiveness
CRSwNP: Chronic rhinosinusitis with nasal polyps
BEC: Blood eosinophil count
FeNO: Exhaled nitric oxide
FEV1: Forced expiratory volume in one second
TSLP: Thymic stromal lymphopoietin
